# COVID-19: experiences of lockdown and support needs in children and young adults with kidney conditions

**DOI:** 10.1007/s00467-021-05041-8

**Published:** 2021-03-19

**Authors:** Yincent Tse, Anne-Sophie E. Darlington, Kay Tyerman, Dean Wallace, Tanya Pankhurst, Sofia Chantziara, David Culliford, Alejandra Recio-Saucedo, Arvind Nagra

**Affiliations:** 1 Great North Children’s Hospital, Newcastle Upon Tyne, UK; 2 School of Health Sciences, University of Southampton, Southampton, UK; 3 Department of Paediatric Nephrology, Leeds Children’s Hospital, Leeds, UK; 4 Department of Paediatric Nephrology, Royal Manchester Children’s Hospital, Manchester, UK; 5 University Hospitals Birmingham NHS Foundation Trust, Edgbaston, Birmingham, UK; 6 NIHR Applied Research Collaboration Wessex, School of Health Sciences, University of Southampton, Southampton, UK; 7 Faculty of Medicine, University of Southampton, Southampton, UK; 8 Southampton Children’s Hospital, Southampton, UK

**Keywords:** Age, Paediatrics, Quality of life, COVID-19

## Abstract

**Background:**

During the initial COVID-19 pandemic, young United Kingdom (UK) kidney patients underwent lockdown and those with increased vulnerabilities socially isolated or ‘shielded’ at home. The experiences, information needs, decision-making and support needs of children and young adult (CYA) patients or their parents during this period is not well known.

**Methods:**

A UK-wide online survey co-produced with patients was conducted in May 2020 amongst CYA aged 12–30, or parents of children aged < 18 years with any long-term kidney condition. Participants answered qualitative open text alongside quantitative closed questions. Thematic content analysis using a three-stage coding process was conducted.

**Results:**

One-hundred and eighteen CYA (median age 21) and 197 parents of children (median age 10) responded. Predominant concerns from CYA were heightened vigilance about viral (68%) and kidney symptoms (77%) and detrimental impact on education or work opportunities (70%). Parents feared the virus more than CYA (71% vs. 40%), and had concerns that their child would catch the virus from them (64%) and would have an adverse impact on other children at home (65%). CYA thematic analysis revealed strong belief of becoming seriously ill if they contracted COVID-19; lost educational opportunities, socialisation and career development; and frustration with the public for not following social distancing rules. Positive outcomes included improved family relationships and community cohesion. Only a minority (14–21% CYA and 20–31% parents, merged questions) desired more support. Subgroup analysis identified greater negative psychological impact in the shielded group.

**Conclusions:**

This survey demonstrates substantial concern and need for accurate tailored advice for CYA based on individualised risks to improve shared decision making.

**Graphical abstract:**

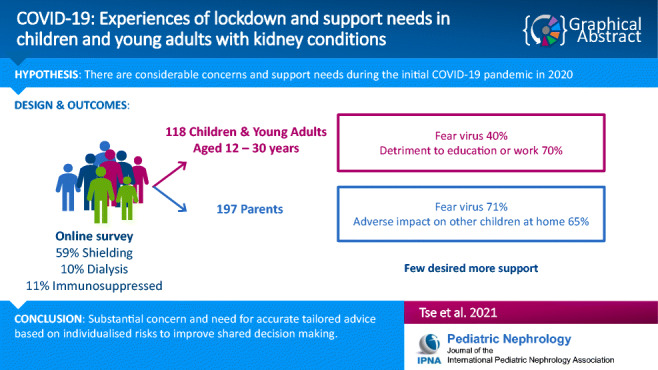

**Supplementary Information:**

The online version contains supplementary material available at 10.1007/s00467-021-05041-8.

## Introduction

On 23 March 2020, in response to the COVID-19 pandemic, the UK entered a continuous period of ‘lockdown’, with closure of schools, workplaces and restrictions on outdoor movements other than exercise or essential shopping. Studies into the experiences of families under quarantine for previous pandemics (SARS-CoV-1, MERS) and in Chinese adolescents during COVID-19 showed very high levels of traumatic distress [1, 2]. Prior to the current pandemic, children and young adults (CYA) with chronic kidney diseases (CKD) already had well-established psycho-social vulnerabilities; most studies reported a greater rate of social and behavioural problems with adjustment disorders, depression, anxiety and neurocognitive disorders compared to their peers [3–5]. They were less likely to be in a relationship, more likely to live in the family home, receive no income, be unable to work due to ill-health and have worse well-being than peers in the general population [6, 7].

Early during the UK epidemic, the government issued a list of conditions and associated treatment regimens considered to constitute extreme clinical vulnerability to COVID-19 disease [8]. For kidney patients, regardless of age, these initially included those requiring dialysis, or on immunosuppression, e.g. kidney transplant recipients and non-transplant indications at an arbitrary level which changed several times during the pandemic. They were recommended to ‘shield’, i.e. to remain at home at all times, and have no face-to-face contact with anyone outside of their household [9].

During this period of prolonged lockdown, it became evident that the potential harms of COVID-19 (the disease caused by the virus SARS-CoV-2) to CYA even with severe co-morbidities were not only from the virus itself but also from being kept at home and away from friends, education or fledgling careers [1]. Fortunately, in stark contrast to the devastating 31% mortality rate in the over 40-year age kidney transplant recipients, there were no deaths reported in under 40-year-olds [10]. The impact of missing education in CYA with CKD who already have lower cognition compared with the general population is unknown, and there is also significant concern about the gaps in safeguarding protection when vulnerable children are not visible to teachers [11, 12].

Little is known about the concerns and decision-making in this young patient group, especially given the rapidly evolving instructions in the early stages of the pandemic. Existing professional networks of charities, clinicians, academics, parents, young patients and young adults were mobilised to develop this study with the aims of increasing our understanding of the evolving experiences, information needs and decision-making in CYA and parents of children living with a kidney condition.

## Methods

### Study design

A survey of CYA with a kidney condition (12–30 years) and parents with children aged 0–18 years who have kidney conditions, assessing experiences, information and support needs and decision-making was undertaken. The survey was co-produced in its design, piloting and dissemination by CYA, parents, clinicians, youth workers and patient advocacy groups. Due to time pressure and socially distancing regulations, CYA representatives were recruited from personal contacts and video focus groups held to refine survey questions before dissemination.

The survey opened between 11 May and 1 June 2020, during the initial wave (including lockdown) of the COVID-19 pandemic within the UK. On 1 June we closed the survey to coincide with the first significant UK easing of lockdown reopening schools and permitting people to meet outdoors. The study was approved by University of Southampton and UK NHS Health Research Authority Research Ethics Committees (IRAS nr. 282176).

Whilst SARS-CoV-2 refers to the virus and COVID-19 refers to the clinical disease, during co-design, we found that this distinction was not appreciated by many non-medical people. For this reason, the questionnaires referred to COVID-19 to cover the virus and the disease. For convenience, the term is used in this way throughout the paper.

### Participants

Participants were CYA who self-identified they were affected ‘with any long-term kidney condition’ aged between 12 and 30 years, or parents/guardian of a child aged between 0 and 18 years, able to read and respond in English. Participants were recruited from across the UK by healthcare teams, national kidney charities, targeted closed Facebook groups and social media. The survey was available either as a link or via poster QR code accessible on a PC, tablet or smartphone. Electronic consent was obtained before completing the online survey. Up to 200 respondents (parents) and 100 young people were intended to be recruited to ensure sufficient numbers of participants to map the range of issues and experiences [13], identify common issues across them, carry out meaningful subgroup analyses and provide rich data from the open text qualitative responses [14, 15].

### Survey

The core set of questions was based on currently available literature [16–18], expert clinician input, co-produced with CYA and parents of a child with cancer (*n* = 7) and then adapted by parents and CYA with a kidney condition (*n* = 13). Further refinement was performed over two video-conference focus groups with a small number of CYA charity group representatives (*n* = 3) who clarified any contentious questions with their groups to come up with the final survey. We sought opinion on content, phrasing, usability and value of each question for research. The survey consisted of two sections consisting of (a) qualitative open questions and (b) quantitative closed statements.

Seven open questions were asked: Experiences: *Can you tell us about your experiences and views on the virus in relation to your child with a kidney condition?*; Hospital*: I worry the hospital is no longer a safe place during the virus outbreak—if so in what way?*; Positive aspects: *I have experienced some positive things to come out of the virus outbreak, either at home or related to care, if so in what way?*; Mood: *In relation to the virus outbreak, my mood or behaviour has changed, if so in what way?;* Information: *Can you tell us where you get information on the virus and what other information you might need?*; Decisions: *Can you tell us how you make decisions about looking after your child in relation to the virus?*; and Support: *What additional support would you like, at home or in hospital, in relation to the virus?*. These open questions were intended to be completed prior to the closed statements which guided the respondent’s thinking. A final free text question asked respondents whether they had any further comments.

The closed statement items were in the following domains: Experiences (*n* = 13), Information (*n* = 7), Decisions (*n* = 10) and Support needs (*n* = 5). A small number of items differed between the parent and CYA surveys. Response options were *Not at all, A little, Quite a bit and Very much* (except for two conditional questions with *Yes*/*No* as response options). For simplicity, COVID-19 was referred to as ‘the virus’. Demographic information was also collected, such as age and treatment.

### Data analysis

The qualitative open questions data were subjected to a thematic content analysis, informed by a three-stage coding process [19, 20]: Stage 1: Initial samples were open-coded into broad comment categories by two researchers (SC, AR), refining an existing framework, and resolving any conflicts with a third researcher (ASD); Stage 2: The framework was used to categorise all comments from the data, with further refinement; and Stage 3: Overarching themes were identified from analysis of similarities in the content between categories. Number of comments were counted, to identify weight of themes. Because of the overlap in comments to categories, the total number of comments does not match the number of participants. Illustrative quotes that most represented each subtheme were chosen by consensus (SC, AR, ASD).

Descriptive statistics were carried out using IBM Statistical Package for Social Science (SPSS) to summarise the demographic data for the two participant groups, and undertake simple descriptive statistics of the closed statement items (collapsing the lowest two response options (Not at all, A little) and the highest two response options (Quite a bit, Very much) into a binary outcome). Subgroup analyses were carried out on an item level, using chi-squared analyses at the two-sided significance level *p* < 0.05, according to whether young people were in the shielded group (instruction from UK Government to stay isolated) or not, separately for the CYA and parent samples. No subgroup analyses were carried out according to age.

## Results

### Participants

One-hundred and eighteen CYA with kidney conditions answered the survey, median age was 21 years (range 12–30 years) with 40 (33.9%) under 18 years old. Respondents’ ethnicities were similar to age-matched prevalent Renal Registry patient cohort with 96 (81.4%) identifying as white.

One-hundred and ninety-seven parents completed the survey, the majority mothers (*n* = 169, 85.8%) and of white ethnicity (*n* = 186, 94.4%). The median age of the children of responding parents was 10 years (range 0–18 years). Table 1 summarises respondents’ characteristics.

**Table 1:** Respondent characteristics

Surveys	Children and young Adults (CYA)	Parents/guardian
Completed by, *n*	118	197
Participant age, years, median (range)	21 (12–30)	41 (21–60)
Child’s age, years, median (range)		10 (0–18)
Ethnicity, *n* (%)		
White	96 (81.4%)	186 (94.4%)
Asian	9 (7.6%)	6 (3.0%)
Black	3 (2.5%)	0
Mixed	6 (5.1%)	3 (1.5%)
Other	3 (2.5%)	2 (1.0%)
Missing	1 (0.8 %)	0
CYA in shielded group, *n* (%)		
Yes No	73 (61.9%) 31 (26.3%)	113 (57.4%) 78 (39.6%)
Do not know	12 (10.2%)	6 (3.0%)
Missing	2 (1.7%)	0
CYA treatment, *n* (%)		
Dialysis at home	4 (3.4%)	9 (4.6%)
Dialysis in hospital	12 (10.2%)	8 (4.1%)
Immunosuppression	10 (8.5%)	25 (12.7%)
No treatment	28 (23.7%)	40 (20.3%)
Other treatment	22 (18.6%)	59 (29.9%)
Missing	42 (35.6%)	56 (28.4%)

### Quantitative results from survey responses

**Fig. 1:**
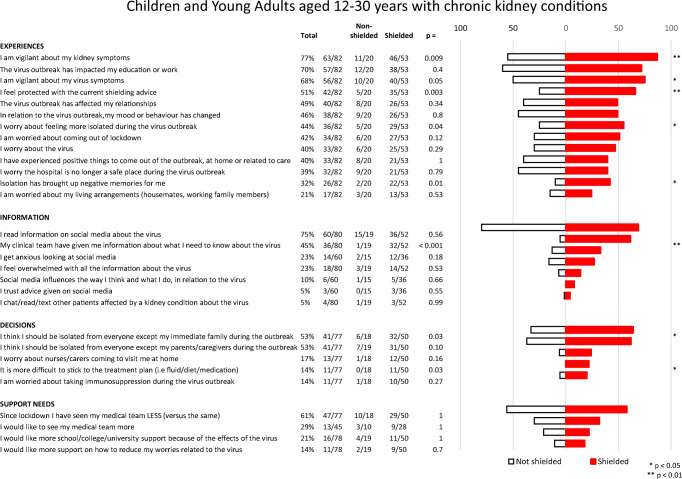
Children and young adults aged 12–30 years with chronic kidney conditions. Amalgamated responses of those who answered ‘quite a bit’ or ‘very much’ to each question

**Fig. 2:**
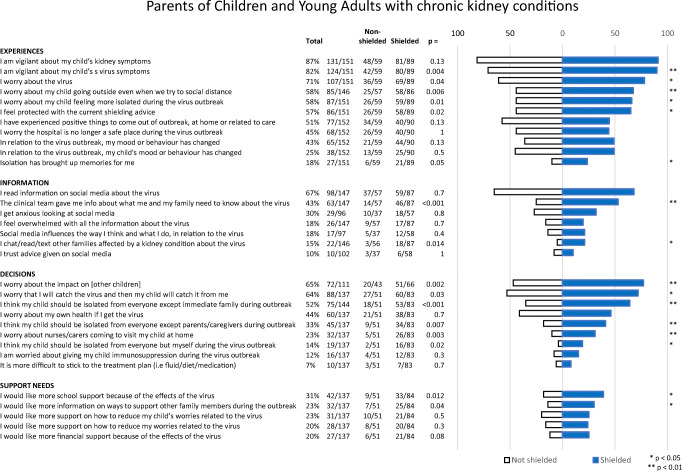
Parents of children and young adults aged 12–30 years with chronic kidney conditions. Amalgamated responses of those who answered ‘quite a bit’ or ‘very much’ to each question

The study generated quantitative results from the closed statement items, which are presented as those who responded with ‘Quite a bit’ or ‘Very much’ for both CYA (Fig. 1) and parents (Fig. 2). Subgroup analyses, comparing those who were shielded with those who were not, are also included.

The majority of CYA were vigilant about virus symptoms (68%) or ‘kidney’ symptoms (77%), whilst 40% worried about the virus. For 70% of CYA, education and work had been impacted. Information came from their clinical team (45%) or through accessing social media (75%). Sixty-one percent of CYA reported being seen less by their clinical team compared to before the pandemic.

Most parents worried about the virus (72%), or were vigilant about virus symptoms (83%) or kidney symptoms (87%). Over half of the parents (57%) felt protected with the shielding advice. Information came from clinical teams (43%) but more from social media (67%). A third of those who accessed information through social media were left feeling anxious (31%). Parents worried that their child would catch the virus from them (65%) and worried about the impact on other children at home (66%). Only a small minority reported that they would like more support from an educational institution (CYA 14%, parents 20%) or support to reduce worries (CYA 21%, parents 31%).

### Shielded vs. non-shielded patients

Results of subgroup analyses that assessed differences between the shielded and non-shielded groups are summarised in Figs. 1 and 2. Over half of CYA, either responding themselves (62%) or included by parental response (57%), were in the shielded group—they recalled receiving a letter or text message from the government. A small number of respondents (10%) were unaware if they were shielded or not. There was no perceived difference between the groups in whether they saw their clinical teams less during the lockdown period (shielded 43% vs. non-shielded 35%, *p* = 0.47).

For CYA, significant differences between shielded and non-shielded groups were found in 8 out of 29 (28%) questions in all domains except support needs. Shielded CYA reported being more vigilant about virus symptoms (76% vs. 50%, *p* = 0.05) and kidney symptoms (87% vs. 55%, *p* = 0.009), more worried about feeling isolated (55% vs. 25%, *p* = 0.035), more likely that isolation brought up negative memories (73% vs. 27%, *p* = 0.01) and felt more protected with shielding advice (66% vs. 25%, *p* = 0.003). Shielded CYA felt that they received adequate information from their clinical teams (62% vs. 5%, *p* < 0.001), should be isolated (64% vs. 33%, *p* = 0.03) and stated that it was more difficult to adhere to their treatment plans (22% vs. 0%, *p* = 0.03).

For parents, shielded vs. non-shielded group differences were found in 16 out of 32 (50%) questions throughout all domains. To highlight the 7 most significant differences (*p* < 0.01), parents of shielded children were significantly more vigilant about virus symptoms (90% vs. 71%, *p* = 0.004) and worried about their child going outside (67% vs. 44%, *p* = 0.006). They reported better information from their clinical teams (53% vs. 25%, *p* = 0.001). In the decisions domain more parents thought their child should be isolated to their immediate families (64% vs. 35%, *p* = 0.001) or just their parents/caregivers (41% vs. 18%, *p* = 0.007); they worried more about home visits from nurses/caregivers (*p* = 31% vs. 10%, *p* = 0.006) and worried more about the impact on other children in the family (77% vs. 47%, *p* = 0.002).

### Qualitative findings from open text questions

Qualitative open questions contained a total of 1,406 quotes (228 Experiences, 158 Hospital, 165 Positive aspects, 262 Mood, 171 Information, 214 Decision making and 208 Support). There was considerable overlap in answers given, and it was observed that participants, after answering the first question about their Experiences, were repeating their responses in the remaining open questions.

Thematic content analyses with example quotes from the Experiences question are presented in Table 2. Five main themes emerged concerning the virus (risk of infection, information guidance and advice and change in healthcare provision) or about lockdown and isolation (the psychological and social impact and keeping safe).

**Table 2:** Qualitative findings from open questions under experiences

Theme	Subtheme	Number of quotes		Quotes	
		CYA	Parent	CYA	Parent
Virus					
Risk of infection	Concerns over getting ill and taking extra precautions to shield.	21	65	The only thing that worries me really is knowing the fact if I got it, it would mean most likely another hospital admission.	We started to shield the week before lockdown due to his kidney and heart condition. We became very concerned about the effect covid-19 is having on the kidneys and with our son already having previous problems and bi duplex kidneys we were almost relieved to be told to shield officially.
	No concerns about the virus. Worries initially but not now.	12	17	I always look after myself so no change.	As she is only at Stage 1 we have not many serious worries. I am very conscious of hygiene and that she drinks her liquids as instructed.
	Concern over visiting hospitals.	6	3	Unfortunately, I am awaiting a scan at the hospital due to my GFR having dropped compared to my normal, and this is something causing me anxiety, especially in the current climate, as I worry about the safety relating to the virus in the hospital.	We have stayed home and not been out, apart from hospital visits and a stay. In hospital is terrifying because you know a lot of people have the virus and have been around the virus and not even stick to social distancing.
	Concerns about other family members contracting the virus.	5	-	My main worry is about my family who have to go to the shops/still work to bring me and my grandparents’ food.	-
	Concerns about the virus entering the home from an infected family member/parent who is a key worker.	-	5	-	As I am still working throughout the lockdown, I worry constantly about contracting the virus and bringing it home to my child and other family members living at home. I work as a customer assistant for Tesco so am in constant contact with other people.
	General expression of fear.	-	5		My child is 7 years old. He was diagnosed two years ago is needing twice a week hospital treatment and daily immunosuppression (tacrolimus, MMF, steroids). For us, COVID has exacerbated our child's anxiety and struggles. He has always coped very well despite the demands of his condition, which is incurable and progressive. Since the COVID pandemic, he has struggled with severe anxieties around dying.
	CYA possibly has had/possibly had COVID.	2	2	Anxious and worried to start with, did my best to self-isolate as it's just me and mother at home. Unfortunately, she caught the virus and me being vulnerable, even though we kept apart and did my best to clean my areas.	It is likely that as a family we caught COVID 19 prior to lockdown (close contact with a confirmed case). The children had a mild cough but were otherwise fine.
Information, guidance and advice	Limited information/mixed messaging.	11	14	I am unsure what level of vulnerability I am, I tried speaking my doctor but heard nothing back or they do not return my calls.	No information has been provided for us other than usual government advice.
	Need for targeted advice and support.	-	4	-	I feel at the beginning we got very little information from the hospital as to what we needed to do. I have taken all my advice so far from the shielding government advice however, I would like to see more specific advice relating to post transplant patients.
	Good information from hospital staff.	-	1	-	I have absolute trust in my daughter’s consultant and have been reassured that she is not at risk because of having kidney disease.
Healthcare provision	Delays regarding kidney transplant operations.	6	3	Very worried that transplant isn’t going ahead because of the virus.	The fact we had a transplant planned which was cancelled due to the virus of course has not been a wonderful experience.
	Miss routine monitors, check-ups.	5	3	My worries are delaying kidney check-ups and the big gaps that are between each appointment during the pandemic.	I feel that since the lockdown people like my daughter have been ignored. She was due to have her quarterly check-up approximately 5 weeks ago and was told not to attend the hospital and they would sort something out about having her bloods checked and this still has not happened.
	New ways of working at the hospital.	2	5	The hospital atmosphere is very different very quiet, clearly taking appropriate procedures to prevent patients getting the virus.	He has had a routine hospital visit in the lockdown period and we were impressed with how safe we felt in the hospital setting.
	Concern over (possible or real) change in rules who can visit the child if in hospital and only one parent able to accompany CYA.	-	3	-	Even further restrictions were put in place and my husband was no longer able to visit. In the following weeks I ended up getting the virus myself and I had to go home to self-isolate (…) When I returned the rules changed quite regularly regarding my husband visiting—I found this quite frustrating as depending on who I spoke to I got a different answer.
Lockdown and isolation					
Psychological and social impact	Missing out on work related and educational opportunities.	14	4	As a student nurse I was offered to opt in to help with the pandemic. Although I am receiving no treatment and just check up, I felt that I was more at risk because of my kidney condition and other health conditions. Therefore, I decided to opt out, which case potentially could impact my degree overall.	She is in a new town and a new college and was starting to try to make some new friends and now that is all back on hold it will be stressful for her starting again. Remote learning is hard at times.
	Missing family and friends.	9	2	Have been unable to visit friends and family including my grandad who is in a care home, so I am worried about him.	Not being able to see friends and family and not being able to go to preschool, especially when he is feeling well enough to go.
	Experiencing more restrictions and missing out on life compared to their peers.	8	-	I feel very locked out of the world, like an outsider, as I hear about everyone else going to the shops and going on walks shows the world is changing.	-
	Separation from partners.	4	-	I made the decision to change my living arrangements and move back full time to live with my parents, as my dad is retired and my mum has been told she can work from home for as long as I need to shield. I normally split my time between living with my parents, and my partner and his family. It was felt it would be inappropriate to stay with my partner as he works in a school (a key worker), and his mum in a GP surgery (also a key worker). We felt, for this reason, it was safer for me to return home. This has been challenging, emotionally, but we are getting through it	
	Delayed resumption of normality after treatment.	2	-	I am also down about having to shield, as after so many years of dealing with lupus and kidney failure, I now have my transplant and feel well, yet must stay away from others to stay well which feels isolating like when I had to dialyse and miss out on my masters nights outs**.**	
	Impractical nature of social distancing	-	2	-	Impossible to follow full shielding advise in small family home.
Keeping safe under lockdown	Concern once restrictions are lifted/adjustment concerns.	7	6	I am worried about the easing of the lockdown as my work will start to make arrangements for me to come back, my work is a front line receptionist at a University so we do have daily contact with people. I will have to move back to my partners if I go back to work which is another risky situation as his parents work at supermarkets so could catch the virus and give it to me.	We live in a small village so it's quite easy to isolate and can still exercise in the garden and ride bikes. I will probably be more anxious when we stop shielding.
	Concern over societal compliance in social distancing and delayed lockdown.	7	-	I was not too worried to start with, I kept to social distancing and handwashing, but it was other people not following advice that was starting to worry me. So now as much as possible I am staying indoors as much as I can.	-
	Difficulty securing provisions (food, cleaning, medication).	2	1	I want my tacrolimus to be available locally at pharmacies not only hospital.	Extremely difficult to access delivery for food/medicines/essentials due to our rural location and have become reliant on charitable help.
	Reliance on friends and family to pick up provisions.	1	1	I mainly avoid going out and since I live with my mom, she usually does the essential shopping.	My daughter does have good friends that live nearby who are dropping off shopping for her and picking up her prescriptions as we live 2½ hours drive away.
	Being in lockdown keeps CYA safe	**-**	2	-	We are very frustrated by the lockdown but understand why we need to do it.
	Priority/lack of priority	1	-	Getting to grips with shielding and signing up for government support (mainly for priority food shopping) was tricky but luckily Kidney Care UK put some guidance together to help once it was made clearer which category we fell under.	

Examples of representative quotes from the subthemes are displayed. *N* = number of quotes. Numbers do not correspond with the ones provided in the section Qualitative Findings as comments were often broken down and categorised under a number of different themes and subthemes

The strongest signal from both CYA (*n* = 21) and parents (*n* = 65) related to the concern that contracting the virus would lead to serious illness and they were taking extra precautions to shield. However, this was not universal; a sizable number of comments from CYA (*n* = 12) and parents (*n* = 17) stated they had no concerns about the virus or had initial concerns which subsided. The predominant comments from CYA (*n* = 11) and parents (*n* = 14) in the information, guidance and advice subtheme, felt they had received only limited information or mixed messages.

When sub-themed by psychological and social impact, CYA (*n* = 37) generated many more comments compared to parents (*n* = 8). The most common comments from CYA was they felt they were missing out on work-related and educational opportunities (*n* = 14), missing family and friends (*n* = 9) and compared to their peers they lived with more restrictions and were missing out on life (*n* = 8).

In the keeping safe under lockdown subtheme, CYA (*n* = 7) and parents (*n* = 6) were worried about adjusting to life after the lockdown. Several CYA (*n* = 7) were also concerned about the general public not following social distancing and hygiene rules, thus putting everyone at risk.

Findings from the other six open questions are summarised in Table 3.

**Table 3:** Qualitative findings from remaining open questions

Questions and themes	CYA (*N*)	Parents (*N*)
Hospital: I worry the hospital is no longer a safe place during the virus outbreak		
May come into contact with infected patients/staff/visitors	21	37
Most likely to catch the virus at the hospital	15	37
No social distancing with people not following hygiene rules	-	26
Confident that my hospital is taking measures	5	6
Not confident that hospitals are taking measures	4	2
Avoid going to the hospital unless necessary	4	-
Worry I may pass on the virus to vulnerable people	2	-
Positive aspects: I have experienced some positive things to come out of the virus outbreak		
Spending time with family/better relationships with family members	37	80
Community coming together	12	22
Changes in the wider society	4	11
Appreciating simple things	1	8
Mood: In relation to the virus outbreak, my mood or behaviour has changed, if so in what way?		
*Mood amongst CYAs and parents—self report*		
Frustrated/less motivation	35	41
More stress and anxiety	12	52
More relaxed	6	16
Depressed/low mood	-	12
Tired of juggling many responsibilities	-	11
Motivated	1	-
*Mood amongst CYAs—parental report*		
Bored/frustrated	-	28
Missing friends	-	22
Enjoying positive aspects/adjusting well	-	15
Stressed/anxious	-	11
Depression/low mood	-	9
Change of eating habits		2
Information: Where do you get information on the virus and what other information may you need?		
*Source of information*		
Media	48	83
NHS (hospital team/website/GP)	27	53
Government (daily briefings, government website, letters and texts)	25	53
Charities	8	31
Social media	14	14
Internet—not specified	5	19
Family members and friends	14	6
Conduct own research/WHO/CDC	5	8
Workplace/school	2	7
*Additional information required*		
More information on shielding	4	7
How virus effects children with CKD	-	6
Targeted information from hospital	-	4
Statistics of COVID-19 on children with CKD	-	1
Length of time lockdown is expected	2	-
Advice on those waiting for a transplant	2	-
Advice tailored to kidney patients	1	-
Efficacy of masks	1	-
Help available (financial; shopping)	-	-
Decision making: How do you make decisions about looking after yourself/your child in relation to the virus?		
Common sense	32	53
Deciding to shield	20	26
Hospital team/NHS	9	32
Government	6	27
Own knowledge/family and friends	12	19
Decisions are child-led	-	7
I do not make decisions	1	-
Social media	1	-
News	-	1
Charities	-	1
Support: What additional support would you like?		
No additional support required	39	53
More relevant information/more guidelines for those shielding	12	13
Mental health support	7	10
Support from hospital	6	7
Assistance to adjust to life after lockdown	1	8
Financial/employment/education	4	4
How to keep the child safe	-	8
Access to literature and relevant data	-	8
More appointment remotely/access to medication	2	3
Positive news/general reassurance	1	4
Support with home schooling	-	4
Availability of food delivery slots	1	2
Reassurance about the safety of hospitals	2	2
Testing of family members/PPE at home	2	2

Thematic content analysis distilled the themes. Numbers do not necessary correspond with the numbers of respondents or number of questions as quotes were often broken down and categorised under a number of different themes

*N* = number of quotes

## Discussion

This is the first study specifically surveying CYA with kidney conditions and their parents’ experience of the COVID-19 pandemic during lockdown. Both CYA and parents were vigilant about the virus and kidney symptoms. Some notable differences exist between the two groups, e.g. CYA worried less about the virus than parents. CYA felt the impact of lockdown most keenly in terms of education and work; parents were particularly worried about their child catching the virus from them and the impact on others at home. Significant differences were found between shielded and non-shielded groups. Information about the virus was gained from multiple sources including social media but they were not always trusted. Free text responses indicated a strong desire for more tailored information to make sensible decisions about their individual risk. We determined that there was a strong sense of pragmatism as there was little demand for extra support and decisions were largely based on common sense.

### Unique challenges of young people with chronic kidney diseases during COVID

COVID-19 affected our patient population very differently according to age. Less than five kidney replacement patients out of a cohort of more than 1,000 children in the UK had a positive COVID-19 test up until mid-July 2020, at completion of our study, and none had serious disease [21]. Similar mild outcome was found in a global study of COVID-19 in children on immunosuppression for kidney diseases [22]. Reassuring data is also available for young adults although these subgroups are not usually made explicit [10]. Our study indicates that the majority of CYA and parents base their decisions on ‘common sense and what they could reasonably do to protect themselves or their child’ and would therefore value further tailored information based on outcome data to make decisions for themselves and their family.

CYA (42%) did report being worried about coming out of lockdown (this question was not specifically asked of parents). This alongside already reported psycho-social vulnerabilities in this group indicate a likely need for increased mental health and well-being support [7]. This will be particularly important during the on-going pandemic as CYA and parents adjust to the changing COVID-19 landscape, particularly regarding their risk status and return to school, further education or the workplace. Recommendations from professional groups can also help inform and reassure CYA and parents as they potentially move from being reclassified as ‘extremely clinically vulnerable’ to being in a ‘not at increased risk group’ [12]. The positive impact of lockdown on health and well-being should also be evaluated and be reflected in healthcare messages and provisions post-lockdown.

In many countries, specific measures are advised for those thought to be at highest risk; in the UK an estimated 2 million clinically extremely vulnerable people were identified and asked to shield. This is the first study with comparable shielded and non-shielded CYA. Differences in several key domains were found, especially in the parent responses, suggesting the amplification of negative experiences within the shielded group. Both CYA and parents in the shielded group worried about feeling isolated. CYA who were shielded reported feeling restricted, not being able to see friends and family, which also led to mood changes such as anxiety and ‘feeling depressed’. Similar findings were found in a cohort of Chinese children on kidney replacement therapy during lockdown—11% of families reported anxiety and 13% depression [23].

Paradoxically, whilst experiencing negative feelings around isolation, CYA and parents recognised its role in keeping them safe and valued being shielded. This was evident in the findings from the free text responses where CYA reported not being too worried at first, but increasingly feeling concerned about other people not following social distancing rules.

### Strengths and limitation of study

This study has several strengths. It is a national study, co-produced with parents and CYA, representing their voices across a wide range of kidney issues during the pandemic. It is the first study that has directly asked children and young adults living with a kidney condition about the effects of COVID-19 and lockdown. Qualitative research can provide valuable insights into patient and parent experiences and inform health care workers about where to target resources. CYA respondents but not parent respondents were ethnically representative of the UK Renal Registry (UKRR) population [24].

Limitations of the study include the contracted time period in which it was necessarily undertaken and the inability to triangulate data between CYA and their respective parent for those < 19 years or explore themes in depth with targeted interviews or user groups. The self-reporting nature of the survey precluded verification of medical background, treatment, shielding category or adherence to public health recommendations. We did not mandate answers to any of the qualitative or quantitative questions, including shielding categories, resulting in missing data increasingly more prevalent later in the survey. The patients were a heterogeneous group covering the whole spectrum of disease severity. We did not measure access to virtual clinics or urban/rural geography which may have influenced respondents’ experience of healthcare. The use of social media to distribute and access the study may have also limited access to English-speaking individuals with compatible devices/smartphones [25].

### Application of this study

This study offers clinicians managing chronic disease information about how CYA and parents perceive risk and how they access information and support during a pandemic. The study indicated that CYA and parents want clear tailored information about individual risk from those that they trust, their clinical team, and accessing trusted information via social media is preferred. In a recent analysis only two thirds of cancer-related social media information was accurate, so debunking COVID-19 misinformation is a challenge for healthcare teams and governments [26, 27]. Improved information and guidance will also be needed to reassure CYA and parents that they can now access healthcare safely. Our suggestion to help address gaps in information and support needs based on study responses is listed in Table 4.

**Table 4:** Suggestions to help address gap in information and support needs based on study responses

Survey respondents feel:	Healthcare team recommendations
Lack of information about individualised COVID risk and shielding information	Shared decision making with kidney team based on up-to-date data Develop kidney-specific information with specialists on charity websites and social media, both for young people and parents
Worry about hospital visits	Show patients how we have made kidney clinic and dialysis unit safe Make it easier to obtain medicines
Support patients with their worries	Offer psychological support when needed Promote peer-support groups Ask about well-being at consultations
Option to contact their kidney team using technology	Offer video or telephone consultations, and ability to email or text kidney team Develop new ways of finding information, e.g. web forums or online Q&A
Want to build on positive aspects of lockdown (e.g. peer support, technology, family time)	Continue making positive changes even after COVID
Anxiety about emerging from lockdown, education and workplace	Advocating and speaking out for patients

The challenge now will be to keep pace with the fluctuations in virus prevalence and risk, ensure the safe delivery of ongoing care including transplantation and provide support alongside tailored advice to CYA and parents so they can continue to live well during a pandemic.

## Supplementary Information

ESM 1 (PPTX 43 kb)
