# Supplementary material for: COVID-19: experiences of lockdown and support needs in children and young adults with kidney conditions
Source: Pediatr Nephrol. 2021 Mar 19;36(9):2797–810. doi: 10.1007/s00467-021-05041-8 (PMC7979448; doi:10.1007/s00467-021-05041-8)
Supplement: Supplementary file 1 — (PPTX 43 kb) [file 467_2021_5041_MOESM1_ESM.pptx]

## Slide 1
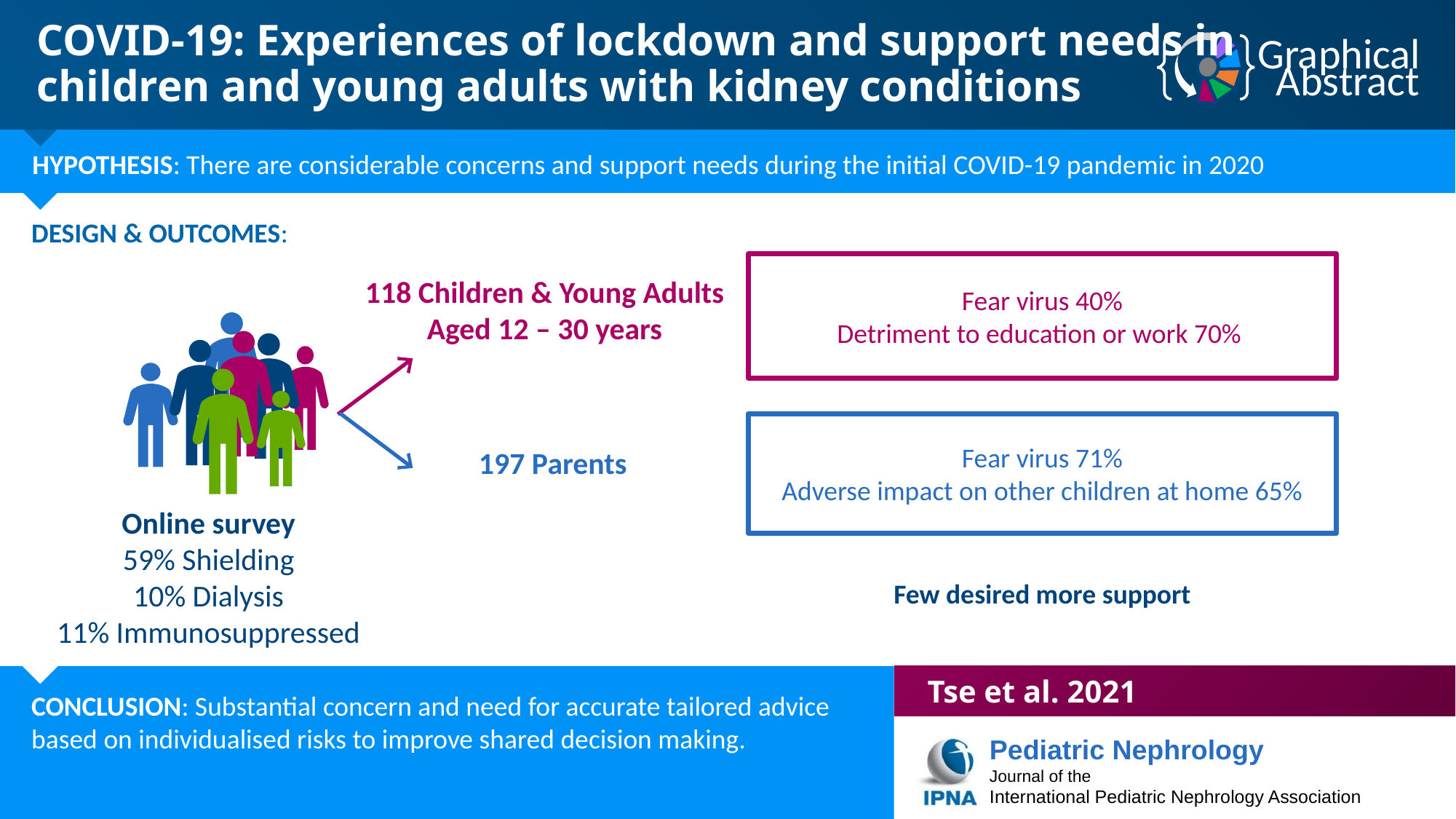

COVID-19: Experiences of lockdown and support needs in
children and young adults with kidney conditions
HYPOTHESIS: There are considerable concerns and support needs during the initial COVID-19 pandemic in 2020
DESIGN & OUTCOMES:
Fear virus 40%
Detriment to education or work 70%
118 Children & Young Adults
Aged 12 – 30 years
Fear virus 71%
Adverse impact on other children at home 65%
197 Parents
Online survey
59% Shielding
10% Dialysis
11% Immunosuppressed
Few desired more support
Tse et al. 2021
CONCLUSION: Substantial concern and need for accurate tailored advice based on individualised risks to improve shared decision making.
